# Prevalence of Multidrug-Resistant Tuberculosis and Associated Factors in Ethiopia: A Systematic Review

**DOI:** 10.1155/2018/7104921

**Published:** 2018-04-03

**Authors:** Solomon Weldegebreal Asgedom, Mebrahtu Teweldemedhin, Hailay Gebreyesus

**Affiliations:** ^1^School of Pharmacy, College of Health Sciences, Mekelle University, Mek'ele, Ethiopia; ^2^Unit of Biomedical Science, School of Medicine, College of Health Sciences and Referral Hospital, Aksum University, Aksum, Ethiopia; ^3^Unit of Health Education and Promotion, College of Health Sciences and Referral Hospital, Aksum University, Aksum, Ethiopia

## Abstract

**Background:**

Multidrug-resistant tuberculosis (MDR-TB) has continued to be a challenge for tuberculosis (TB) control globally. Ethiopia is one of the countries with high MDR-TB burden.

**Objective:**

The main purpose of this study was to determine the prevalence of MDR-TB and associated factors in Ethiopia.

**Methods:**

A systematic review of the literatures on prevalence of MDR-TB and associated factors was conducted in the country.

**Results:**

In our electronic search, 546 citations were depicted. Among the total 546 citations described, a total of 22 articles met eligibility criteria and were included in the review article. According to our review, the prevalence of MDR-TB ranged from 0 to 46.3%. The average mean rate of MDR-TB in Ethiopia was found to be 12.6 ± 15.9%. The overall prevalence of MDR-TB in all TB cases was estimated to be 1.4%. From a total of 3849 patients studied, 527 had MDR-TB. Previous exposure to antituberculosis treatment was the most commonly identified risk factor of MDR-TB in Ethiopia.

**Conclusion:**

Despite relative decline in incidence of MDR-TB, the distribution and prevalence of MDR-TB continued to be a serious challenge for TB control in Ethiopia. Previous exposure to antituberculosis treatment was also the most common risk factor for MDR-TB. Therefore, strong TB and MDR-TB treatment along with tight introduction of follow-up strategies should be applied for better TB control.

## 1. Introduction

Tuberculosis (TB) still continues to be a big public health problem worldwide. It is the second leading cause of death from all infectious diseases globally. Tuberculosis kills an estimate of 1.3 million people every year worldwide [1]. It is also one of the most serious public health challenges in Ethiopia. Ethiopia is ranked second after Nigeria and seventh globally among the 22 countries with high TB burden [2].

The main new barrier that challenges the control of TB is high burden of multidrug-resistant TB (MDR-TB). MDR-TB is a man-made problem due to poor management and quality of antituberculosis drugs. Thus, MDR-TB can be minimized by making tight identification of its predictors [1]. The major contributing factor identified for the spread of MDR-TB is poor infection control [1]. MDR-TB kills an estimate of 110,000 individuals every year and nearly half million new cases of MDR-TB emerge every year. Among the newly emerging MDR-TB cases, only 3% get serious treatment globally [3].

In 2008, there were 440,000 new MDR-TB cases and 150,000 deaths worldwide [4]. According to the Ethiopian national TB drug resistance surveillance reported, 2.3% of new TB cases and 17.8% of previously treated TB cases were estimated to have MDR-TB [5]. In Africa, there was a report of 69,000 MDR-TB cases [4]. Ethiopia ranked 15th with new cases of MDR-TB each year and is one of the 27 countries with high MDR-TB burden [3]. Even if MDR-TB is highly prevalent in retreated TB cases, the prevalence of MDR-TB in newly diagnosed TB patients has been reported to be 2.8% [1, 6]. There are a number of published studies on MDR-TB available worldwide. However, accurate data on MDR-TB in Ethiopia is scarce. This review provides an updated and comprehensive status of the MDR-TB epidemics in Ethiopia. Thus, the main aim of this review was to assess the current prevalence of MDR-TB and factors associated with MDR-TB in Ethiopia.

## 2. Methods

### 2.1. Search Strategy

Studies that estimated rate of MDR-TB and/or identified factors associated with MDR-TB were systematically reviewed. All studies that were published up to 2016 were critically reviewed. We used PubMed search engines and Google Scholar by using medical subject titles MDR-TB, associated factors, and antituberculosis drug resistance. The references of included articles were appropriately scanned to identify additional articles of interest and we used HINARI to access articles without payment.

### 2.2. Selection of Studies

We used the following criteria to include articles in our study:

- Articles with clear objective and methodology
- All articles published up to September 2016
- Articles that address prevalence of MDR-TB and/or associated factors
- Articles published in English language
- Articles with their full texts obtained

### 2.3. Data Collection Tool and Procedure

Data was collected from the articles through development of data collection checklist. The data collection checklist was pretested in 5 randomly selected articles and it was amended accordingly. The checklist included title, authors' names, year of publication, study time, study design, sample size, rate of MDR-TB, predictors of MDR-TB, and study setting. The prevalence of MDR-TB and/or associated factors reported in all the selected studies was tallied and assessed.

### 2.4. Operational Definitions

The following definitions related to drug resistance were used [7]:

- MDR-TB: TB caused by strains of* Mycobacterium tuberculosis* which are resistant to at least Isoniazid (INH) and Rifampicin (RMP)

## 3. Results

**Figure 1:**
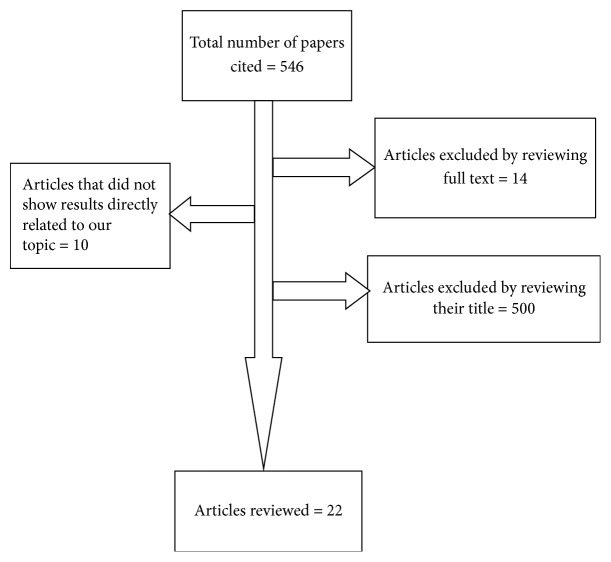
Flow diagram of study articles identification.

In our electronic search, 546 citations were depicted. Among the total 546 articles described, 500 were excluded by reviewing their titles, 14 were excluded following full-text review, and 10 were excluded because the title did not show results directly related to our topic. Finally, a total of 22 articles that met eligibility criteria were included in the review article (Figure 1). Among the total 22 articles that met inclusion criteria, 15 studies only reported the prevalence of MDR-TB and they did not asses the factors that were associated with MDR-TB. On the other hand, seven articles reported factors associated with MDR-TB. Among the 7 articles that assessed predictors of MDR-TB, two articles reported findings exclusively focusing on predictors of MDR-TB. They did not report prevalence of MDR-TB, because all the patients that were included in the study represented exclusively MDR-TB cases.

### 3.1. Prevalence of MDR-TB

In our review, we assessed the overall prevalence of MDR-TB among all TB cases by adding all the number of patients with MDR-TB and dividing it by the total number of studied patients. The total number of patients studied in the review was found to be 3849. Among the total patients studied, 527 had MDR-TB. Thus, the overall prevalence of MDR-TB in this review was found to be 1.4%. The studies were conducted during the period of 1994–2014 from different territories of the country [7–26] (Table 1).

**Figure 2:**
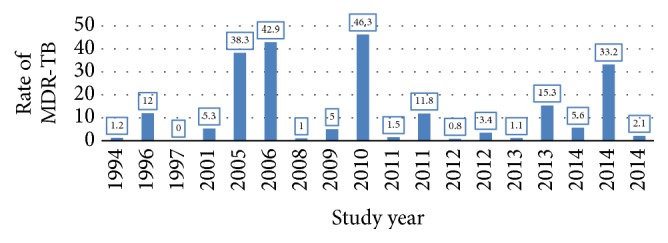
Rate of MDR-TB from 1994 to 2014 in Ethiopia.

The maximum peak rate of MDR-TB recorded in the country was 46.3% in 2010 [16] and the minimum rate of MDR-TB was 0% [7]. The average mean rate of MDR-TB was 12.6 ± 15.9%, ranging from 0 to 46.3% [7–26]. In 2006 and 2005, respectively, second (42.9%) [12] and third (38.3%) maximum peaks of MDR-TB prevalence were reported [11]. Starting from 2011, a significant decline in rate of MDR-TB was found, despite a 33.2% record of MDR-TB in 2014 [18–20, 22, 23, 25, 26] (Table 1). A zero rate of MDR-TB was recorded in Addis Ababa in 1997 [7]. Generally, there were heterogeneous distribution and prevalence rate of MDR-TB report in the country according to the reviewed studies Figure 2.

### 3.2. Risk Factors Associated with MDR-TB

Our review found that the most commonly reported predictor of MDR-TB was previous exposure to anti-TB treatment [12, 14, 15]. On the contrary, Biadglegne et al. in 2014 showed that there was a negative association between MDR-TB and previous exposure to anti-TB treatment [19]. In the study, newly treated TB cases harbor patients from development of MDR-TB [19]. Another risk factor that was found to be a predictor of MDR-TB was HIV disease [13, 17]. Being male has also been identified to be a predictor of MDR-TB in Ethiopia [13, 16, 19]. The factors that were associated with MDR-TB vary from study time to study time and from study place to study place. The different factors that were associated with MDR-TB are described in Table 2.

## 4. Discussion

This study reviewed the prevalence rate of MDR-TB and the different factors associated with MDR-TB in Ethiopia. In the review, a total of 22 articles that assess prevalence of MDR-TB and/or factors associated with MDR-TB were reviewed. The papers were conducted starting from 1994 to 2014. The prevalence rate found as well as the factors associated with MDR-TB was found to be frequently heterogeneous with respect to geographical areas and study periods.

This review study found prevalence of MDR-TB to be 1.4% among all TB cases with an average mean rate of 12.6 ± 15.9%, ranging from 0 to 46.3% [7–26]. This finding is lower as compared to the 2017 WHO anti-TB drug resistance surveillance data report, which showed that 4.1% of new and 19% of previously treated TB cases in the world are estimated to have Rifampicin-resistant or multidrug-resistant tuberculosis (RR-TB/MDR-TB) [29]. This might be due to study design difference, where 2017 MDR-TB WHO report's estimated prevalence was for either Rifampicin resistance or Rifampicin and Isoniazid resistance. However, in our study, resistance to both Rifampicin and Isoniazid was declared as MDR-TB. Moreover, 2017 WHO MDR-TB surveillance report estimated the prevalence of MDR-TB in new TB cases and previously treated cases separately. In our study, we could not estimate prevalence of MDR-TB in new TB cases and retreated TB cases separately due to reported variation of the published papers. Moreover, estimates presented by global control programs are based on samples from government centers comprising potentially susceptible populations or populations where the infection appearance or recurrence is monitored regularly and treated optimally. Therefore, estimates generated from an analysis of these samples may not be a true representation of the TB population in the real world [30, 31]. An Indian systematic review also found MDR-TB prevalence of 23.3% among all TB cases [32]. This is quite higher when compared to our study. This might be due to variation in patient group studied. The healthcare setup, MDR-TB detection, and the population socioeconomic status variation between the two countries might contribute to the discrepancy.

The lowest record of MDR-TB was in 1997 [7], whereas the highest record of MDR-TB was in 2010 [16]. This highest record of MDR-TB in 2010 as compared to earlier periods might be due to previous antituberculosis exposure of TB cases. Starting from 2010, relatively significant decline in MDR-TB was reported [18–20, 22, 23, 25, 26]. This relative decline in MDR-TB could be due to great emphasis given for quality provision of TB control and diagnostic parameters in the country. The country achieved not only TB control but also MDR-TB treatment of ≥75% in 2015, making the country one of the three countries with high MDR-TB burden which achieved MDR-TB treatment of ≥75% [33]. This showed that the control of TB and treatment of MDR-TB are escalating from time to time in Ethiopia. Despite improvement in incidence of MDR-TB, this finding showed the spread of MDR-TB and the local control measures for the prevention of MDR-TB are still unsatisfactory.

The commonly identified risk factor of MDR-TB in this review was previous exposure of patients to antituberculosis treatment [12, 14, 15, 24–27, 33]. Previous antituberculosis treatment exposure is also a common risk factor for MDR-TB in many other countries like India, Spain, Iran, Portugal, Europe, and other east African countries [34–41]. The WHO report in 2013 also confirmed that the highest prevalence of MDR-TB among new and previously treated TB cases was 3.6% and 20.2%, respectively, which is higher in previously treated patients. The high association of previous TB treatment and MDR-TB might be explained due to inappropriate chemotherapy regimens, inadequate or irregular drug supply, unsatisfactory patient or clinician compliance, lack of supervision of treatment, and absence of infection control measures in hospitals [37]. Another factor that was commonly identified as a risk factor was HIV/AIDS [13–15, 17, 19]. In addition, alcohol use and treatment failure also were found to be predictors of MDR-TB in Ethiopia [24, 27]. This is in congruence with the finding depicted from Spain [42]. Being male and young age were also other factors that were described as determinant factors of MDR-TB in Ethiopia [16, 19, 24]. However, there were also a number of reports from the country (Ethiopia) which contradict the finding [12, 14, 15]. The discrepancy could be due to difference in study subjects, sample size, and study variables studied.

In this study, there are some limitations. The number of articles that were reviewed was very small due to scarcity of published articles from the country. In the reviewed articles, the study designs used were heterogeneous, which could bias the finding. There was also a discrepancy in reporting of findings. Some studies only studied rate of MDR-TB; other studies studied only factors associated with MDR-TB. In this review study, there was a heterogeneous report of the articles outcome.

## 5. Conclusion

Even if the incidence of MDR-TB was declined relatively, MDR-TB prevalence and distribution is still a serious public health problem in the country. Prior history of antituberculosis treatment was the most commonly identified risk factor for MDR-TB. Thus, improving TB follow-up strategy in association with provision of strong primary TB as well as MDR-TB treatment could improve TB control in Ethiopia.

## Figures and Tables

**Table 1:** Prevalence of MDR-TB in Ethiopia in the period of 1994–2014.

Author	Number of subjects	Study time/period	MDR-TB
Dmissie et al. [7]			
New cases	167	1994	2 (1.2%)
Retreated cases	NS		NS
Total	167		2 (1.2%)
Ahmed and Hoffner [8]			
New cases	NS	1996	NS
Retreated cases	107		13 (12%)
Total	107		13 (12%)
Bruchfeld et al. [9]			
New cases	103		0
Retreated cases	18	1997	0
Total	121		0
Eyob et al. [10]			
New cases	73		2 (2.7%)
Retreated cases	19	2001	2 (10.5%)
Not known	2		1
Total	94		5 (5.3%)
Dest et al. [11]			
New cases	73		46 (38.3%)
Retreated cases	NS	2005	NS
Total	73		46 (38.3%)
Agonafi et al. [12]			
New cases	44		1 (2.3%)
Retreated cases	63	2006	45 (71.4%)
Total	114		46 (42.9%)
Yimer et al. [13]			
New cases	93		1 (1%)
Retreated cases	NS	2008	NS
Total	93		1 (1%)
Abebe et al. [14]			
New cases	214		8 (4.2%)
Retreated cases	46	2009	5 (10.9%)
Total	260		13 (5%)
Abate et al. [15]			
New cases	NR		NR
Retreated cases	NR	2010	NR
Total	376		174 (46.3%)
Abate et al. [16]			
New cases	NR		NR
Retreated cases	NR	2010-2011	NR
Total	136		2 (1.5%)
Tessema et al. [17]			
New cases	136		2 (1.5%)
Retreated cases	NS	2011	NS
Total	136		2 (1.5%)
Hussein et al. [18]			
New cases	93		11 (11.7%)
Retreated cases	9	2011	1 (11.11%)
Total	102		12 (11.8%)
Biadglegne et al. [19]			
New cases	212	2012	1 (0.5%)
Retreated cases	13		0
Total	225		2 (0.8%)
Adane et al. [20]			
New cases	77		1 (1.29%)
Retreated cases	12	2012	2 (18.66%)
Total	89		3 (3.37%)
Nigus et al. [21]			
New cases	NR		NR
Retreated cases	NR	2012-2013	NR
Total	606		93 (15.3%)
Shegaw [22]			
New cases	NR		NR
Retreated cases	NR	2012–2014	NR
Total	434		9 (2.1%)
Seyoum et al. [23]			
New cases	357		4 (1.1)
Retreated cases	NS	2013	NS
Total	357		4 (1.1%)
Mulisa et al. [24]			
New cases	NR		NR
Retreated cases	NR	2013-2014	NR
Total	265		88 (33.2%)
Hamusse et al. [25]			
New cases	85		2 (2.4%)
Retreated cases	21	2013-2014	3 (14.3%)
Total	106		5 (4.2%)
Mekonnen et al. [26]			
New cases	88		2 (2.3%)
Retreated cases	36	2014	5 (13.9%)
Total	124		7 (5.6%)

MDR-TB: multidrug-resistant tuberculosis; NR: not reported; NS: not studied.

**Table 2:** Risk factors associated with MDR-TB in Ethiopia from 1994 to 2014.

Risk factors	Negative association with MDR-TB	Positive association with MDR-TB
Previous exposure to anti-TB treatment	Biadglegne et al. [19]	Agonafi et al. [12], Abate et al. [15], Abebe et al. [14], Nigus et al. [21], Mulisa et al. [24], Hamusse et al. [25], and Mekonnen et al. [26]
TB history of defaulter	NR	Nigus et al. [21]
HIV/AIDS	Abate et al. [15] and Abebe et al. [14]	Yimer et al. [13], Tessema et al. [17], and Mulisa et al. [24]
Age	Yimer et al. [13], Abate et al. [15], Biadglegne et al. [19], and Abebe et al. [14]	Nigus et al. [21] and Mulu et al. [27]
Being male	Yimer et al. [13], Abate et al. [15], and Abebe et al. [14]	Biadglegne et al. [19], Mulisa et al. [24], and Abate et al. [16]
Occupation (farmer)	NR	Mulisa et al. [24]
Known TB contact history	NR	Mulisa et al. [24]
Nonadherence to previous TB treatment	NR	Shegaw [22]
History of previous TB treatment	NR	Mulisa et al. [24], Shegaw [22], and Deressa and Demissie [28]
Opportunistic infection	NR	Deressa and Demissie [28]
Newly treated cases	Agonafir et al. [12], Abate et al. [15], and Abebe et al. [14]	Mulu et al. [27]
Lack of formal education	NR	Mulisa et al. [24]
Low monthly income	NR	Mulu et al. [27]
Cavitation on chest X-ray	NR	Mulu et al. [27]
History of contact with MDR-TB	NR	Mulu et al. [27]
Rural residence	NR	Mulisa et al. [24]
Alcohol use	NR	Mulisa et al. [24] and Mulu et al. [27]
Chat chew	NR	Mulisa et al. [24]
Previous unfavorable TB treatment outcome	NR	Mulisa et al. [24]
Previous history of TB treatment failure	NR	Mulu et al. [27]
Primary education	NR	Deressa and Demissie [28]
Unemployment	NR	Deressa and Demissie [28]
Long distance to the healthcare facility	NR	Deressa and Demissie [28]

NR: not reported.

## Data Availability

The datasets supporting the conclusions of the study are included in the article. Any additional data will be available on request.

## Abbreviations

HIV: Human immunodeficiency virus
MDR-TB: Multidrug-resistant tuberculosis
TB: Tuberculosis.
